# Toward Bioelectronic Medicine—Neuromodulation of Small Peripheral Nerves Using Flexible Neural Clip

**DOI:** 10.1002/advs.201700149

**Published:** 2017-07-26

**Authors:** Sanghoon Lee, Wendy Yen Xian Peh, Jiahui Wang, Fengyuan Yang, John S. Ho, Nitish V. Thakor, Shih‐Cheng Yen, Chengkuo Lee

**Affiliations:** ^1^ Department of Electrical and Computer Engineering National University of Singapore 4 Engineering Drive 3 Singapore 117583 Singapore; ^2^ Singapore Institute for Neurotechnology (SINAPSE) National University of Singapore 28 Medical Drive, #05‐COR Singapore 117456 Singapore; ^3^ Center for Intelligent Sensors and MEMS National University of Singapore 4 Engineering Drive 3 Singapore 117576 Singapore; ^4^ NUS Suzhou Research Institute (NUSRI) Industrial Park Suzhou 215123 P. R. China; ^5^ Graduate School for Integrative Science and Engineering National University of Singapore Singapore 117456 Singapore; ^6^ Department of Biomedical Engineering School of Medicine Johns Hopkins University Baltimore MD 21205 USA

**Keywords:** bioelectronic medicine, neural clips, neuromodulation, pelvic nerve, vagus nerve

## Abstract

Neural modulation technology and the capability to affect organ function have spawned the new field of bioelectronic medicine. Therapeutic interventions depend on wireless bioelectronic neural interfaces that can conformally and easily attach to small (few hundred micrometers) nerves located deep in the body without neural damage. Besides size, factors like flexibility and compliance to attach and adapt to visceral nerves associated moving organs are of paramount importance and have not been previously addressed. This study proposes a novel flexible neural clip (FNC) that can be used to interface with a variety of different peripheral nerves. To illustrate the flexibility of the design, this study stimulates the pelvic nerve, the vagus nerve, and branches of the sciatic nerve and evaluates the feasibility of the design in modulating the function of each of these nerves. It is found that this FNC allows fine‐tuning of physiological processes such as micturition, heart rate, and muscle contractions. Furthermore, this study also tests the ability of wirelessly powered FNC to enable remote modulation of visceral pelvic nerves located deep in the body. These results show that the FNC can be used with a range of different nerves, providing one of the critical pieces in the field of bioelectronics medicines.

## 1 Introduction

The emerging field of bioelectronics involves monitoring and modulating biological signals to control the bodily functions and to treat diseases. With the remarkable improvement in soft and stretchable devices that enable the integration of a variety of biosensors and wireless powering elements, e‐skin, and wearable technologies have shown promising prospects of advanced health care.^1^ Recently, the target of interest is moving from the human skin to the internal organs of the body by combining these cutting‐edge technologies with neuroscience, enabling bioelectronic medicine.^2^, ^3^ Such implantable bioelectronics, which requires a biocompatible, miniature, soft, and wirelessly powered platform^4^ may interface with not only the central nervous systems (CNS) for restoring or substituting neourological deficits or disabilities,^5^ but the peripheral nervous systems (PNS) to control neuroprostheses,^6^, ^7^, ^8^ or physiological functions.^2^, ^9^ For this, neural interfacing technology (NIT), which provides the basis for direct communication with neuron tissues and mapping neural signals, is an essential area to be preferentially developed among principal research areas in bioelectronic medicine.^3^ In case of NIT in the CNS applications, glial scar‐free neural recordings have been achieved using ultraflexible probes in the brains of rodents.^10^ Furthermore, the brain–spine interface was able to support the locomotion of monkeys using a multimodal neural interface, called e‐dura.^11^, ^12^

For the PNS, extra‐neural (cuff and FINE)^6^ and intra‐fascicular (TIME)^7^ interfaces were implanted on sensory nerves in the hands of human subjects to provide sensory feedback from prosthetic arms. However, the intra‐fascicular approach itself may be invasive leading to nerve damage as well as difficulty in implantation on small visceral nerves.^13^ For the use of extra‐neural cuff electrodes, the current sizes need to be scaled for small visceral nerves.^3^ This is limited in that the fabrication of cuff electrodes typically requires significant manual assembly of relatively bulky cuff and lead wires. This also makes high‐quality mass production challenging and makes integration of the electrodes with active components for wireless transmission rather difficult. Furthermore, as a result of the need to match the diameter of the nerves precisely, it is often necessary to prepare a number of cuffs of different sizes for each implantation. Helix electrodes have been used for vagus nerve stimulation (VNS) in humans as a new treatment modality for epilepsy and drug‐resistant depression, although there are limitations in challenging surgical procedures of implantations of helical coil electrode, anchoring tether, and the lead due to the narrow surgical place.^14^, ^15^ Even though new extraneural approaches with flexible materials have recently shown promising results for peripheral nerve modulation,^16^ those are limited in surgical implantation on small visceral nerves due to its small size and the narrow space with movement in the viscera.

To achieve NIT for bioelectronic medicine, new paradigm‐shift approach must be adapted and miniaturized enough to interrogate visceral nerves wirelessly and securely.^3^ Here are grand challenges due to many physiological and anatomical difficulties in accessing deep nerves associated with the autonomic nervous system. These challenges include (i) for the autonomic nervous system, complex innervation of the organs or muscles, rendering precise control of specific functions challenging; (ii) quick and mechanically secure implantation which is an important consideration in the presence of physiological motion such as respiratory and cardiovascular movements; and (iii) considerable compliance and flexibility from the neural interfaces since nerves are highly compliant and associated with moving organs. Additionally, the integration of NIT with wireless powering by either ultrasound^17^ or electromagnetic powers^18^ is a promising direction for future bioelectronic medicine.

**Figure 1:**
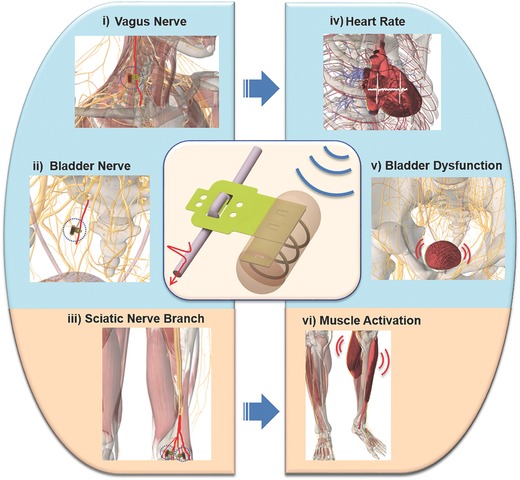
Schematic diagram of peripheral nerves and modulated functions using wireless flexible neural clip (FNC). Schematic diagram of different applications of a wireless FNC interface for wireless modulation of nerves to achieve different organ or tissue output. (i) Vagus nerve stimulation (VNS), (ii) bladder nerve stimulation, and (iii) the stimulation of sciatic nerve branches for modulation of (iv) heart rate (HR), (v) bladder dysfunction, and (vi) leg muscles, respectively.

**Figure 2:**
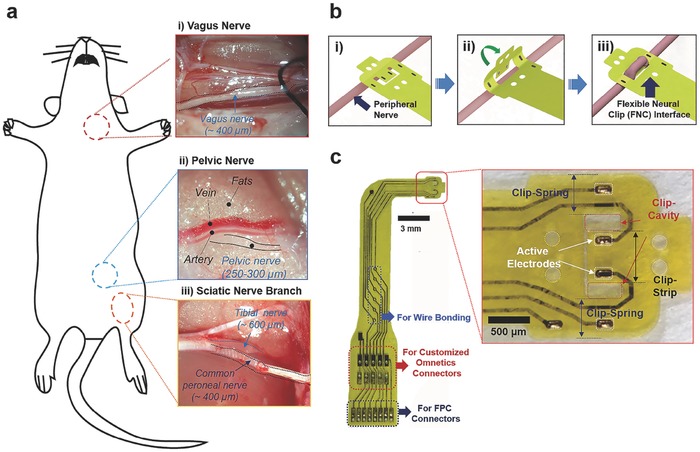
Experimental setup and flexible neural clip (FNC) interface. a) Photomicrographs of small peripheral nerves in rats; (i) a vagus nerve, (ii) a pelvic nerve, and (iii) sciatic nerve branches. b) Schematic diagram of the steps involved in implanting the FNC on a peripheral nerve. The FNC can be implanted onto a peripheral nerve easily by inserting the nerve between the clip‐strip and clip‐springs after slightly bending the clip‐springs (i–iii). c) Photomicrographs of the fabricated FNC and (inset) clip‐head. The FNC also has various interfaces applicable for wire bonding, customized Omnetics, and FPC connectors.

Here, we propose a flexible neural clip (FNC) implant that enables not only easy and conformal implantation on a variety of small peripheral nerves but remote modulation. This novel implant is able to support the development of wireless neural interface for bioelectronic medicine applications. We targeted three nerves that required small neural interfaces for modulating the specific physiological functions of organs or tissues. They are the vagus nerve (**Figure** **1**‐i), the bladder nerve (Figure 1‐ii), and the sciatic nerve branches (Figure 1‐iii). The modulation of vagus and bladder pelvic nerves leads to the promising potential of achieving restoration of homeostatic physiological conditions of patients.^19^, ^20^, ^21^, ^22^, ^23^ We investigated heart rate (HR) modulation (Figure 1‐iv), bladder function control (Figure 1‐v), and leg muscle contraction (Figure 1‐vi) with the aids of our novel FNC interface by stimulating these nerves using rats as the animal model in this study (**Figure** **2**a‐i–iii). Furthermore, active FNC as a form of wireless neural dust was also developed to provide batteryless remote modulation of bladder function.

## 2 Results

### 2.1 Interface Design

Our novel FNC interface enables easy and conformal implantation on a variety of the peripheral nerves in a manner analogous to clipping a paper clip. The FNC can be implanted onto a peripheral nerve easily by inserting the nerve between the clip‐strip and clip‐springs after slightly bending the clip‐springs (Figure 2b). This ease of implantation has great surgical benefits when the FNC is implanted in small nerves located inside the body in narrow and deep spaces, accompanied by continuous movements such as respiration and heartbeat. This interface provides not only conformal contact with the nerve but also gentle pressure on the nerve to keep the clip interface in place. For this unique functionality, we determined the clip dimensions (length, width, and thickness of the clip‐springs, the clip‐strip, and the clip‐cavities) (inset of Figure 2c) by considering the size of different peripheral nerves and the material properties of polyimide after conducting several implantation tests on the nerves. The FNC is also designed to be easily scalable and integrated with other implantable components (Figure 2c; details of FNC design is described in Figure S1, Supporting Information). The size of the clip‐cavity for CP nerves and vagus nerves (diameter: ≈400 µm) was 700 µm × 500 µm, and the clip strip was 650 µm × 900 µm. The width of the clip‐spring was the same as the length of cavity (700 µm) to maintain the spring elasticity of the polyimide. Two active electrodes (each 17672 µm^2^) were located on the center of the clip‐strip with the distance of 350 µm for reliable position and stimulation of the implanted nerve. This FNC can cover bigger (diameter: ≈600 µm) or smaller sizes (diameter: ≈300 µm) of nerves owing to the functionality of the clip design during acute *in vivo* test. We also designed the miniature FNC interface for pelvic nerves (diameter: 250–300 µm). The size of the clip‐cavity was 470 µm × 200 µm and the clip strip was 420 µm × 650 µm. The width of the clip‐spring was also the same as the length of cavity (470 µm). The size of active electrode was 13024 µm^2^ and the distance between the electrodes was 400 µm. This design allows reliable implantation and stimulation of pelvic nerves in rats. The detailed fabrication procedures are described in the Experimental section and Figure S2 (Supporting Information).

Electrochemical interface is of paramount importance for stimulation. High electrode impedances will require high voltages to inject desired current amplitudes, which results in undesirable electrochemical reactions, possibly damaging both the electrode and the tissue. If the charge delivery capacity is poor, high current amplitudes will be required for the activation of the nerve, which may also cause nerve damage or delamination of the electrode surface.^24^

Iridium oxide is widely used due to its good stability and large charge storage capacity (CSC) for neural recording and stimulation. Among three typical fabrication methods; sputtering iridium oxide film, activated oxide film, and electrodeposited iridium oxide film (EIROF), EIROF shows the largest CSC and lowest impedance.^25^ Adherent electrodeposited films are most easily obtained on Au substrate compared with other noble metals such as Pt and PtIr.^26^ To enhance electrochemical characteristics of stimulation, the released electrodes were coated with EIROF. The coated IrO_2_ on Au sensing electrodes showed a good impedance value (1.9 ± 0.09 kΩ at 1 kHz, *n* = 10), and a cathodic charge storage capacity (56.4 ± 2.42 mC cm^−2^, *n* = 10) for stimulation. These values are comparable or even better to materials used previously in the literature for neural stimulation.^25^, ^26^, ^27^ This result demonstrates that the IrO_2_‐coated electrodes can be used for in vivo stimulation experiments. The detailed procedures of the coating method and the electrochemical characterization are described in the Experimental section as well as the result of electrochemical characterization is in Figure S3 (Supporting Information).

### 2.2 Stimulation of Pelvic Nerve for Modulation of Bladder Function

**Figure 3:**
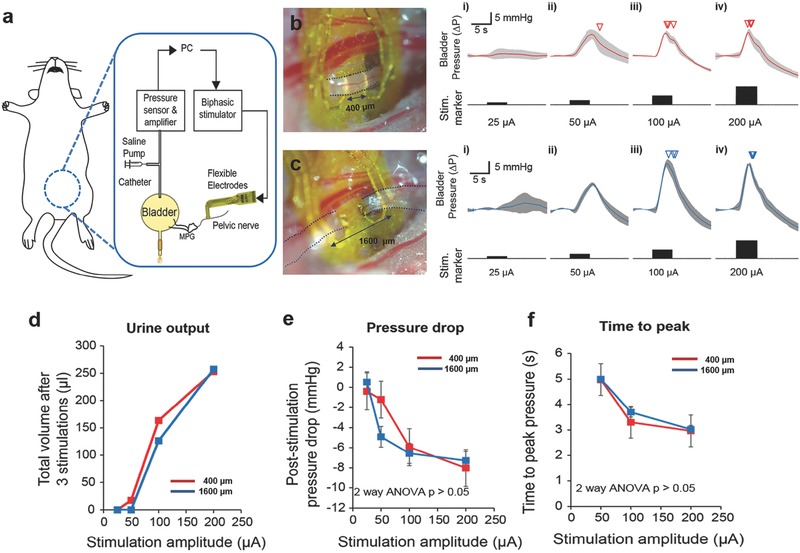
Pelvic nerve stimulation to modulate bladder function. a) Schematic diagram of pelvic nerve stimulation for the modulation of bladder function. Evoked intrabladder pressure changes and micturition outcome with increasing stimulation amplitudes for b) short (400 µm) and c) long interlead distances (1600 µm). Photomicrographs of implanted flexible neural clip (FNC) electrodes on the same nerve in two different configurations are shown in (b) and (c). Increases in bladder pressure due to pelvic nerve stimulation was observed for both implantation configurations at increasing stimulation current from (i) 25 µA, (ii) 50 µA, (iii) 100 µA, and (iv) 200 µA, respectively (*n* = 3 trials). Inverted triangles denote the onset of voiding events. d) Urine output as a result of different stimulation amplitudes. e) Poststimulation pressure drops as a result of different stimulation amplitudes. f) Time to reach peak pressure as a result of different stimulation amplitudes. At suprathreshold amplitudes, the poststimulation pressure drop, and time to reach a peak in bladder pressure were similar and not significantly different between the two implantation configurations (two‐way ANOVA, *p* > 0.05, *n* = 3 trials each).

Bladder dysfunction, which remains a major healthcare challenge and can impair daily life of patients, is a widely studied area in the field of neuromodulation.^28^ The pelvic nerve is a promising stimulation target for the control of bladder function as it provides autonomic efferent inputs to contract the bladder detrusor muscle, and is anatomically and functionally more specific for bladder neuromodulation.^19^, ^28^, ^29^ However, the pelvic nerve is a small visceral nerve located deep within the body, which leads to difficulties in implantation, as well as maintaining contact for reliable stimulation if current neural electrodes are used.^6^, ^12^, ^13^, ^30^ We successfully performed stimulation of the pelvic nerves using a miniature FNC to control bladder function while monitoring bladder pressure (**Figure** **3**a). The FNC interfaced successfully with the pelvic nerve in two different implantation configurations: either 400 µm (Figure 3b) or 1600 µm (Figure 3c) interelectrode site distances. Bladder contraction corresponding to increased positive bladder pressure changes was observed for both implantation configurations at increasing simulation currents (range: 25–200 µA, *n* = 3 trials). Lower “subthreshold” stimulation amplitudes increased bladder pressures without micturition, while higher “suprathreshold” amplitude (100–200 µA) caused larger pressure changes and led to graded and repeatable miturition of urine. At suprathreshold amplitudes, the urine output (Figure 3d), poststimulation pressure drop (Figure 3e), and time to reach peak bladder pressures (Figure 3f) were similar and not significantly different between the two implantation configurations, indicating that functional, consistent electrode–nerve interfaces were achieved. The results suggest that (1) the FNC can reliably be implanted onto small pelvic nerves while maintaining effective electrical contact with the nerve for reliable and repeatable stimulation in *in vivo* anesthetized situations, and (2) the FNC is mechanically robust to withstand handling across different experiments. The present findings indicate that the pelvic nerve was stimulated in a graded manner to control bladder contractions, leading to reproducible micturition events comparable with other published techniques that use large size electrodes with high possibility of damaging nerves along the usage process.^21^, ^31^

### 2.3 Stimulation of Vagus Nerve for Controlling Heart Rate

The Food and Drug Administration (FDA) approved VNS as an adjunctive, nonpharmacological therapy for patients with medically refractory partial onset seizures in 1997, as well as an adjunctive long‐term therapy for chronic or recurrent major depression in 2005.^32^ Furthermore, VNS has shown other clinical benefits in diseases such as hypertension and rheumatoid arthritis.^14^, ^22^, ^33^ Recently, VNS has been proposed as a promising therapeutic approach such as heart failure^34^ and cardiac arrhythmia^35^ as it provides cardiac autonomic responses such as chronotropic, inotropic, and dromotropic effects.^23^, ^33^, ^36^ For the clinical applications, several VNS factors such as pulse width, current amplitude, number of pulses, interpulse period, and electrode configurations still need to be verified with the physiological effects, though the therapeutic effect has been recognized in preclinical and pilot clinical studies.^23^ While helical or cuff‐type electrodes are commonly used for larger sized vagus nerves,^14^, ^37^ implanting these electrodes on smaller nerves are still challenging due to the cylindrical shape and encircling mechanism for good electrode–nerve contact. This is a technical challenge that has to be addressed especially for chronic epilepsy studies of common small animal models such as mice and rats.^38^

**Figure 4:**
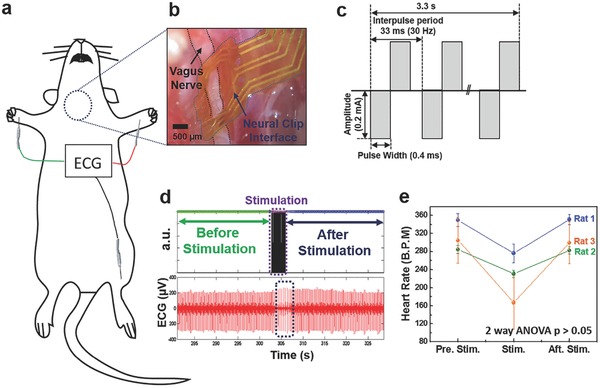
Vagus nerve stimulation (VNS) to control heart rate (HR). a) Schematic diagram of VNS for the control of HR. b) A photomicrograph of an implanted flexible neural clip (FNC) on a vagus nerve in a rat. c) Schematic diagram of biphasic pulses for the stimulation. d) Electrocardiogram (ECG) recordings before and after VNS. e) The change in HR caused by VNS (two‐way ANOVA, *p* < 0.05). Circles indicate the mean value and bars represent the standard error of the mean.

In this study, we demonstrated that the FNC, with its flatter profile and clip mechanism for achieving contact with nerves, is a novel way to interface with the vagus nerve in rodents. We performed VNS in rats using the FNC to control HR while monitoring the electrocardiogram (ECG) (**Figure** **4**a). The FNC was implanted easily on the vagus nerve (Figure 4b), despite the continuous movement of the nerve due to respiration and heartbeats. First, we conducted preliminary tests with a range of stimulation amplitudes and pulse widths derived from a previous study.^33^ Afterward, we repeated VNS with one parameter in multiple rats (*n* = 3) with the parameter (30 Hz, pulse width of 0.4 ms, duration of 3.3 s, and amplitude of 0.2 mA) (Figure 4c). The HR was calculated by interbeat interval (RR interval), which represent chronotropic effect.^23^ The HR was significantly reduced during the stimulation, and recovered to baseline after the stimulation (Figure 4d). The mean reduction in the HR was 25.5% during the stimulation, and the HR recovered to 99.8% (*n* = 17) of the HR before stimulation when it was measured at 10 min after the stimulation was stopped (Figure 4e). The result indicates that the HR changes are comparable or slightly more effective than previous studies^33^, ^39^ within the safety range of the stimulation parameter, though it cannot directly be compared with other studies due to complex stimulation parameters and experimental conditions. The efficacy of our small FNC in visceral vagus neuromodulation was proved.

### 2.4 Stimulation of Sciatic Nerve Branch for Muscle Activation

**Figure 5:**
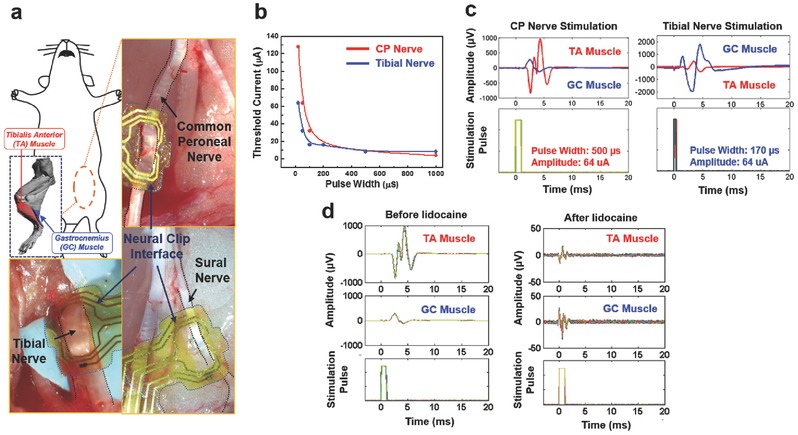
Stimulation of sciatic nerve branches to control muscles. a) Schematic diagram and photomicrographs of sciatic nerve branches. b) Threshold currents versus pulse widths when stimulating the common peroneal (CP) and tibial nerves. The biphasic pulse widths were varied between 20 and 1000 µs. The *I*_rh_ of the CP nerve was 4 µA, and the calculated *T*_ch_ from the curve was 470 µs. For the tibial nerve, the *I*_rh_ was 8 µA, and the *T*_ch_ was 180 µs. c) The recorded electromyogram (EMG) signals and stimulation pulses of the CP and tibial nerve stimulation. d) The recorded EMG signals and stimulation pulses of the CP nerve stimulation before lidocaine application (left panel) and after lidocaine application (right panel).

We tested the FNC on various sciatic branches as the size of the branches is comparable to visceral nerves and serves as an additional test bed to interrogate the peripheral limb system. In addition, the specificity of the sciatic nerve branches (i.e., the common peroneal (CP) nerve primarily innervates the tibialis anterior (TA) muscle that controls ankle dorsiflexion^40^ while the tibial nerve mainly activates the gastrocnemius (GC) muscle for ankle extension^41^) make them ideal for showcasing the function of the FNC. We stimulated the CP nerve, tibial nerve, and sural nerve in rats (**Figure** **5**a) while recording compound muscle action potentials (CMAPs) from the GC and the TA muscles. To optimize the effectiveness of stimulation, we sought to determine the chronxie (*T*_ch_ ) that is the shortest pulse width at a current amplitude about twice of the rheobase current (*I*_rh_).^42^ We measured the threshold current needed to evoke the CMAPs as a function of pulse width for the CP nerve and the tibial nerve (Figure 5b). After that, we demonstrated the CP nerve and tibial nerve stimulation using pulse widths close to their respective *T*_ch_, (CP nerve: 500 µs; tibial nerve: 170 µs) but at current amplitudes higher than the respective thresholds (Figure 5c). The TA muscle was activated more than the GC muscle during CP nerve stimulation, and ankle dorsiflexion was clearly observed. During tibial nerve stimulation, the GC muscle was activated more than the TA muscle, and ankle extension (plantar‐flexion) with leg stretch was visible. To verify whether the muscle activation was a result of the nerve stimulation, we applied lidocaine for blocking nerve function^43^ to the CP nerve and conducted the same stimulation procedure again after 10 min (Figure 5d). The CP nerve was blocked completely, and no stimulation‐evoked electromyogram (EMG) signals were observed. We also conducted the sural nerve stimulation. However, only small twitch of the middle toe in the rat was observed and there were no muscle signals of the TA and the GC, a result that is consistent with previous literature showing that sural nerve mainly includes sensory fibers instead of motor fibers that activate the muscle.^44^ These results indicate that the FNC work well on sciatic nerve branches with different sizes, and consequently can be used to evoke different patterns of muscle activation. Furthermore, we stimulated the sciatic branches with corresponding *T*_ch_ that provides the most energy‐efficiency stimulation.^43^ This possibly indicates a paradigm‐shift approach of restoring signals for bionic limb using wireless and multiple FNCs implanted on smaller nerve branches, in contrast to bulky multichannel cuff‐types electrodes required for precise selective stimulation techniques.^6^

### 2.5 Wireless Stimulation of Pelvic Nerve for Modulation of Bladder Function

**Figure 6:**
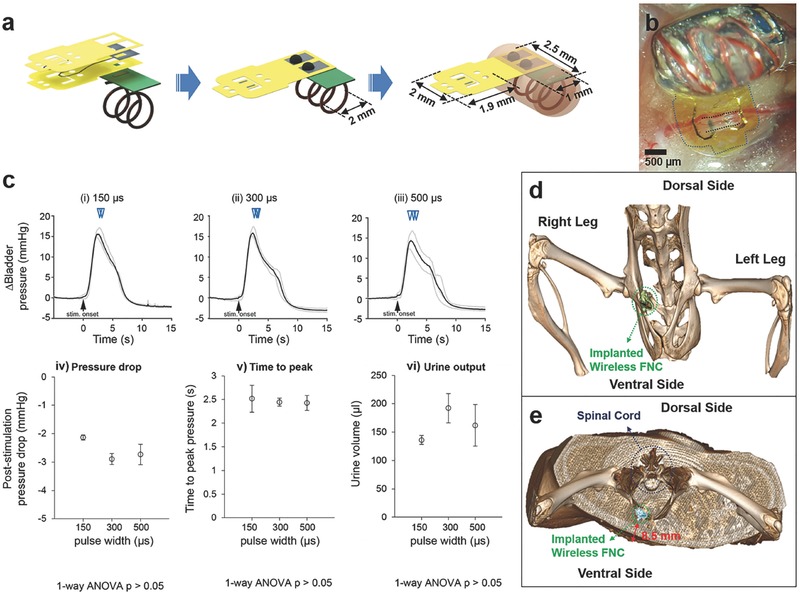
Wireless pelvic nerve stimulation using active flexible neural clip (FNC) interfaces. a) Schematic diagram of the assembly of the active FNC. b) A photomicrograph of the implanted active FNC on a pelvic nerve in a rat. c) Results of the pelvic nerve stimulation on bladder pressure changes, pressure drop, time to reach peak, and urine output as a result of phase width (i) 150 µs, (ii) 300 µs, and (iii) 500 µs, respectively. Actual duration was (i) 5.21 ± 0.21 s, (ii) 6.12 ± 0.58 s, and (iii) 5.86 ± 1.00 s, respectively and the mean was 5.73 ± 0.71 s. Inverted triangles denote the onset of voiding events. d) Micro‐CT image of the implanted FNC; (inset) magnified image of the FNC. e) Cross‐sectional view of the Micro‐CT image.

Many visceral nerves reside deep in the body close to critical organs. Reaching and stimulating these nerves requires excess wiring as well as powering scheme. To avoid implanting a power source, we performed wireless remote neuromodulation of pelvic nerves using the FNC integrated with a tiny coil (**Figure** **6**a) for the midfield powering scheme which allows the transfer of mW levels of power to FNC in deep tissue (>5 cm).^18^ Figure 6b shows the assembled active FNC implanted on a pelvic nerve. First, we employed the same stimulation parameters used in previous pelvic nerve experiments as the first step of trials, and then subsequently we slightly increased the pulse phase from 150 to 500 µs (Figure 6c). At the phase width of 150 µs, overall responses such as normalized pressure and urine volume were relatively stable. The urine output increased when the phase width increased from 150 to 300 µs, but it slightly decreased from 300 to 500 µs, albeit nonsignificantly (one‐way ANOVA, *p* > 0.05, *n* = 3 trials each). The detailed assembly and wireless stimulation procedure are described in Figures S7 and S8 (Supporting Information). To determine the implanted device's distance from the skin, we performed micro‐CT imaging after suturing the abdomen. The small active FNC was positioned well near pubis bone (Figure 6d) and the distance between the implanted active FNC and skin is 8.5 mm (Figure 6e). It indicates that approaching of wireless transfer from ventral side is acceptable or even a preferred alternative due to the short depth of the implantation.

## 3 Discussion

For the bladder nerve application, we stimulated pelvic nerves for the control of bladder function, which is relatively less explored and compared to nerve targets. Part of the reason could be due to the deep abdominal location of the pelvic nerve and respiratory‐related movements of the pelvic area, which demands novel bioelectronic neural interfaces for stable interfacing with thin visceral nerves and that is currently lacking in the field. Notably, we showed that our novel FNC can provide effective electrical contact with the pelvic nerve for reliably and repeatable stimulation in acute *in vivo* anesthetized situations. The availability of the FNC for the pelvic nerve would facilitate more studies in the future on the potential of pelvic nerve stimulation for various bladder dysfunction.

For the vagus nerve application, we performed VNS using the FNC to reduce HR in rats. The FNC could easily be implanted on the vagus nerve despite the continuous movement of the nerve due to respiration and heartbeats. By repeating the VNS with one stimulation parameter in multiple rats, we observed a certain range of reduction of HR that was reasonable or even slightly more effective than previous studies. This result shows that the FNC can provide reliable stimulation on vagus nerves. With a smaller scale of the FNC, such as that used for the pelvic nerve stimulation, the electrode could similarly be implanted onto mouse vagus nerves. Addition of more active leads on the clip strip of the FNC may provide increased spatial specificity to the stimulation. Furthermore, the wireless FNC can have great benefits in chronic epilepsy studies of small animals such as mice and rats in the future.

We approached the sciatic nerve branches using the FNC for effective and selective stimulation to modulate leg muscles instead of main sciatic nerve that is typically accompanied by precise manipulations of a neural interface and selective stimulation technology. Theoretically, we performed the most energy‐efficient stimulation on the branches using the pulse width which is equal to each chronxie (*T*_ch_).^43^ It also indicates that multiple wireless FNCs applied to each most energy‐efficient stimulation parameter for individual target nerves would be effective in improving the stimulus selectivity as well as eliminating the efforts of selective stimulation techniques through the main sciatic nerve.

Lastly, wireless FNC was demonstrated for remote modulation of bladder function in rats. The total size of the wireless FNC was 3 mm × 5 mm, which is significantly smaller size than currently developed neural interfaces and comparable with neural dust (Figure 6a).^17^ This tiny and wirelessly powered FNC could be reliably implanted on the small visceral pelvic nerve deeply located in the body and providing remote modulation of micturition in rats. Furthermore, we confirmed the implantation of the active FNC using micro‐CT imaging, indicating that the depth of wireless transfer was preferred. All results indicate that this wireless FNC is a promising novel neural interface for neuromodulation of visceral nerves.

## 4 Conclusion

The proposed novel FNC shows great potential for use in neuromodulation of small peripheral nerves. In addition, the remote modulation of micturition with a device of several millimeters scale is a significant result that has not yet achieved with current neural interface technologies. This wireless FNC technique will also be applied for the stimulation of vagus nerves or sciatic nerve branches to enable wireless modulation of each function in the future. With future chronic FNC implantation experiments, we can assess nerve health and functionality over time to validate long‐term efficacy and reliability of the FNC. We anticipate our interface does not only provide sciatic nerves interfacing in a paradigm‐shift manner, but also pave a way of doing neural modulation for bioelectronic medicine that requires reliable modulation of small peripheral somatic and visceral nerves, thus advancing implantable bioelectronics toward untapped potential of neuromodulation.

## 5 Experimental Section

*Device Fabrication*: The FNC consisted of a polyimide‐Au‐polyimide sandwiched structure fabricated by microelectromechanical system technology. The fabrication process followed standard photolithographic and clean room procedures. First, a 1 µm thick aluminum (Al) layer was evaporated onto the silicon substrate by physical vapor deposition (Figure S2a, Supporting Information). It acted as a sacrificial layer to release the final device from the substrate. Then a 8 µm base layer of photosensitive polyimide (Durimide 7505, Fujifilm, Japan) was spun onto the Al‐coated substrate with a speed of 2000 rpm (Figure S2b, Supporting Information). After exposure under ultraviolet (UV) light with a dosage of 120 mJ cm^−2^, the base layer was post‐baked and developed in HTRD2 and RER 600 (Fujifilm, Japan), which defined the bottom layer pattern of the FNC. The base polyimide layer was cured at 200 °C in N_2_ for 30 min. In this way, it creates a chemically and physically stable surface for further processing (Figure S2c, Supporting Information). After that, a layer of AZ 9260 (AZ Electronic Materials, USA) was spun onto the polyimide base layer. This AZ layer was exposed and the electrode traces were patterned. A layer of 20 nm chrome (Cr) was deposited to improve the adhesion of the next conduction layer by sputtering. After a 250 nm gold layer was deposited, the conductive metal layer was patterned by a lift‐off process in acetone (Figure S2d, Supporting Information). Another 8 µm top layer of polyimide was spun onto the processed metal layer, and patterned to expose the sensing contacts and connection pads (Figure S2e, Supporting Information). Then, we adopted an anodic metal dissolution approach to release the whole device that not only ensured a flat planar structure was released (Figure S2f, Supporting Information), but also was significantly faster than the traditional wet etching process. Briefly, the wafer was immersed in a 2 m NaCl solution, and connected to an external positive terminal of a voltage source at 1 V. A platinum (Pt) mesh electrode was connected to the negative terminal. A magnetic stir bar was also put inside the solution to keep the concentration of NaCl uniform. After around 20 min, the exposed portions of the Al sacrificial layer were removed, and only the covered portions of the Al sacrificial layer were left. Since the contact area between the Al sacrificial layer and the NaCl solution decreased, the current dropped, and the Al etching rate was reduced. Thus, the voltage was then increased to 20 V to speed up the release process. After the entire Al sacrificial layer was removed after 2 h, the final device was released. The pads of the electrodes can be connected to FPC connectors (Hirose Electric Co. Ltd.), customized Omnetics connectors (Omnetics Connector Corp., MN, USA). Also, the interface can be soldered to any substrate via wire bonding machine (Figure 2c). To enhance electrical stimulation, exposed Au electrodes were electroplated with iridium oxide.

*Iridium Oxide Coating*: The electroplating solution was prepared the modified recipe from the literature^25^, ^26^, ^45^: 300 mg of iridium chloride was dissolved in 200 mL of DI water, and stirred for 15 min. Then, 1000 mg of oxalic acid powder was added to the solution, and stirred for 10 min. Potassium carbonate was slowly added to the solution to adjust the pH to 10.5. The prepared solution was kept at room temperature for 2 d. When it turned into a violet color, it was stored in a dark bottle at 4 °C in the fridge. To electroplate the electrode sites with iridium oxide, a three‐electrode configuration with a silver/silver chloride (Ag/AgCl) electrode, and mesh Pt electrodes for the reference and counter electrodes, was used for initial coating. A triangular voltage pattern of 0.55 V was applied 50 times by a potentiostat (Zennium E, ZAHNER‐elektrik Inc, Germany). Thereafter, the electrode pads were connected to the negative terminal of an external voltage source, and the positive terminal of the external voltage source was connected to a platinum mesh electrode immersed in the solution together. Pulsed voltage, with peak‐to‐peak magnitude of 0.55 V and offset voltage of 0.275 V at 1 Hz was applied for 20 min to plate the iridium oxide.

*Electrochemical Characterization*: To characterize electrochemical performance of the coated electrodes, electrochemical impedance spectroscopy for measuring impedance, as well as cyclic voltammetry for measuring CSC, were carried out using a three‐electrode setup in phosphate buffered saline. A three‐electrode configuration with a silver/silver chloride (Ag/AgCl) electrode, and mesh Pt electrodes for the reference and counter electrodes, was used for both measurements. The output impedance was recorded with an impedance analyzer (Zennium E, ZAHNER‐elektrik Inc, Germany).

*Surgical Implantation*: Adult female Sprague‐Dawley rats (200–300 g) were used for acute *in vivo* experiments in this study. All procedures were performed in accordance with protocols approved by the Institutional Animal Care and Use Committee of the National University of Singapore. The surgery was carried out in accordance with the 143/12 and R15‐0592 protocol. For each experiment, the rat was anesthetized with a mixture (0.2 mL 100 g^−1^) of ketamine (37.5 mg mL^−1^) and xylazine (5 mg mL^−1^) intraperitoneally (I.P.), and supplementary doses of 0.1 mL 100 g^−1^ were injected for maintenance. For the sciatic nerve branch experiment (*N* = 2 rats), after an adequate depth of anesthesia was attained, the right sciatic nerves were exposed through a gluteal‐splitting incision. The FNCs were implanted on the branches. For the vagus nerve experiment (*N* = 3 rats), the right vagus nerves were exposed carefully then, the FNCs were implanted. For the bladder experiment (*N* = 1 rat), the animal was placed in the supine position, and a ventral midline incision of the lower abdomen was first made to expose the bladder and then extended laterally to expose the pelvic nerve. The underlying muscles were cut, and adipose and connective tissues were removed or pushed aside to expose about 2 mm of the nerve for electrode implantation.

*Physiological Characterization of Bladder Functions*: Bladder pressure and urine output were also measured and detected during the stimulation to quantify the functional output. The FNC, which was connected to a stimulator (AM systems 2100) via a FPC connector, was positioned over the nerve using micromanipulators, and “opened” manually using tweezers for the iridium‐oxide coated leads to interface with the nerve at either short or long interactive lead distances. Stimulation parameters were biphasic rectangular waveforms, with a frequency of 10 Hz, 150 µs phase width, duration of 5 s, and amplitudes ranging from 25 to 200 µA. Intrabladder pressure was measured via a saline‐filled catheter (Instech Laboratories Inc.), inserted into the bladder that was connected to a pressure sensor (Transpac IV). An infusion pump for refilling the bladder was used when necessary. A pair of wires (part of a voltage divider circuit) was placed outside the urethral meatus to detect voiding or urine outflow. A data acquisition board (PicoScope 4424) was used to acquire amplified pressure signals, sync pulses from the stimulator, and voltage changes from the urine detection wires at a sampling frequency of 20 KHz. All acquired data were then analyzed using custom MATLAB programs. Pressure data were low‐pass filtered at 30 Hz, and a 5 s period prior to each electrical stimulation was taken as baseline to calculate changes in pressure. The detailed preliminary tests of pelvic nerve stimulation are described in Figures S4–S6 (Supporting Information).

*Wireless Active Neural Clip Interface*: This wireless active FNC (70 mg) consisted of a receiving coil, a rectifier, and an LED. All of them were constructed on a Gold Phoenix printed circuit board (PCB). The coil was wound on top of the PCB with an inner diameter of 2 mm using copper wire (Belden, 36 gauge magnet wire, 200 µm diameter), with three turns depending on the design frequency (≈1.6 GHz). For the rectifier circuit, two Schottky diodes (Skyworks SMS7630‐061) and two 10 pF capacitors were arranged in a one stage rectifier configuration, in which the output voltage is twice the input peak voltage. A LED (Bivar, SM0603UV‐400) was placed after the rectifier to ensure a constant 2.8 V necessary to drive the electrode. The electrode was connected in parallel with the LED. The entire FNC was encapsulated in a silicone elastomer (World Precision Instruments, Kwik‐Sil Adhesive) except the active electrodes of the FNC. The power used to operate the device was around ≈0.1 mW.

## Conflict of Interest

The authors declare no conflict of interest.

## Supporting information

Supplementary
