# Supplementary material for: Toward Bioelectronic Medicine—Neuromodulation of Small Peripheral Nerves Using Flexible Neural Clip
Source: Adv Sci (Weinh). 2017 Jul 26;4(11):1700149. doi: 10.1002/advs.201700149 (PMC5700646; doi:10.1002/advs.201700149)
Supplement: Supplementary file 1 — Supplementary [file ADVS-4-na-s001.pdf]

Copyright WILEY-VCH Verlag GmbH & Co. KGaA, 69469 Weinheim, Germany, 2017.

ADVANCED SCIENCE
Open Access

Supporting Information

for *Adv. Sci.,* DOI: 10.1002/advs.201700149

Toward Bioelectronic Medicine—Neuromodulation of Small Peripheral Nerves Using Flexible Neural Clip

Sanghoon Lee, Wendy Yen Xian Peh, Jiahui Wang, Fengyuan Yang, John S. Ho, Nitish V. Thakor, Shih-Cheng Yen, and Chengkuo Lee**

WILEY-VCH

Copyright WILEY-VCH Verlag GmbH & Co. KGaA, 69469 Weinheim, Germany, 2013.

Supporting Information

Toward Bioelectronic Medicine – Neuromodulation of Small Peripheral Nerves Using Flexible Neural Clip (FNC)

Sanghoon Lee[+], Wendy Yen Xian Peh[+], Jiahui Wang, Fengyuan Yang, John S. Ho, Nitish V. Thakor, Shih-Cheng Yen, and Chengkuo Lee**

S. Lee, J. Wang, F. Yang, Prof. J. S. Ho, Prof. N. V. Thakor, Prof. S. C. Yen and Prof. C. Lee
Department of Electrical & Computer Engineering, National University of Singapore, 4 Engineering Drive 3, 117583, Singapore
Email: shihcheng@nus.edu.sg; elelc@nus.edu.sg

S. Lee, Dr. W. Y. X. Peh, J. Wang, F. Yang, Prof. J. S. Ho, Prof. N. V. Thakor, Prof. S. C. Yen and Prof. C. Lee
Singapore Institute for Neurotechnology (SINAPSE), National University of Singapore, 28 Medical Drive, #05-COR, 117456, Singapore

S. Lee, J. Wang, and Prof. C. Lee
Center for Intelligent Sensors and MEMS, National University of Singapore, 4 Engineering Drive 3, 117576, Singapore
NUS Suzhou Research Institute (NUSRI), Suzhou, Industrial Park, Suzhou, P. R. China 215123

Prof. N. V. Thakor and Prof. C. Lee
Graduate School for Integrative Science and Engineering, National University of Singapore, Singapore 117456

Prof. N. V. Thakor
Department of Biomedical Engineering, School of Medicine, Johns Hopkins University Baltimore, MD 21205, USA

Keywords: bioelectronic medicine, electroceuticals, neural clip, pelvic nerve, vagus nerve, MEMS

[+]These authors contributed equally to this work

***Corresponding Authors:**

Table of Contents

Supplementary Methods **Page**

1. Design of flexible neural clip (FNC) interface 3

2. Preliminary test of pelvic nerve stimulation 3

3. Wireless Neural Clip Interface 4

4. Wireless pelvic nerve stimulation 4

Supplementary Figures and Legends S1-S8 5

References 13

1. Design of flexible neural clip (FNC) interface

A flexible and soft polyimide clip interface provides not only conformal contact with the nerve, but also gentle pressure on the nerve to keep the clip interface in place. The flexible neural clip (FNC) is created based on the design of a paper clip that provides easy and secure implantation. The flexible and biocompatible polyimide serves as a scaffold of the FNC for the functions. The key element of FNC interface is the dimension of clip (length, width, and thickness of clip-springs, clip-strip, and clip-cavities) (**Figure 1**d) considering Young`s modulus of the polyimide of 2.3G Pa.[1] The thickness of the device is largely tradeoff between flexibility and rigidity of the polyimide. To reliably perform clipping and conformal contact to a nerve, the total thickness of 16 μm was experimentally selected. Based on mechanical stress test of clip opening, the acceptable angle (α) between the clip-strip and the clip-spring was around 33~ 34° for reliable and repetitive opening function. The clip-strip width was taken the radius of nerves into account keeping the angle (**Figure S1**a). As shown in **Figure S1**b, this unique design allows gentle pressure to a nerve enough to clip the nerve while still making good contact.

2. Preliminary test of pelvic nerve stimulation

The clip design allowed easy and reliable implantation with less damage and close contacts on the pelvic nerves. We performed repetitive stimulations using supra-threshold amplitudes to show the reproducibility of pelvic nerve stimulation using the FNC (**Figure S4**a; n = 8 trials). Repeatable voiding responses were obtained in all 8 trials during a single continuous recording session, with pressure changes (**Figure S4**b). To examine how reliable our FNC work across different animals, we reused the same FNC for experiments carried out in two rats on different days. Similar to each of the two rats, applying higher stimulation amplitude caused greater peak change in intra-bladder pressures (**Figure S5**a) and decreased the time

taken to reach the peak pressure (**Figure S5**b), indicating that the FNC can be reliably implanted without much lost in electrode performance and is mechanically robust for handling across different experiments.

3. Wireless Neural Clip Interface

Firstly, active version of FNC was fabricated in the same manner of the fabrication procedure where contact pads matched with the size of mini-PCB (**Figure S7**a). Secondly, wireless components including diodes and capacitors were soldered on the PCB (**Figure S7**b). Thirdly, the FNC was aligned on the PCB, then, silver paste was applied for electrical connections. Finally, a coil and UV LED were soldered and the entire FNC was encapsulated in a silicone elastomer except the active electrodes of the FNC.

4. Wireless pelvic nerve stimulation

The prepared wireless FNC was tested in phosphate buffered saline (PBS) solution first before moving to *in vivo* experiments. The wireless FNC was implanted on a pelvic nerve. For consistent repetition of each trial, the wireless external coil was postioned and fixed at a proper place using a manipulator (**Figure S8**a). UV LED was intentionally soldered and extended outside of the wireless FNC to monitor the operation of the device and wireless stimulation (**Figure S8**b). During the stimulation, we made the implantation site dry from body fluids. Stimulation parameters were applied with a frequency of 10 Hz, duration of 5-6 seconds, and phase width of 150, 300, and 500 µs, respectively. Intra-bladder pressure was measured in the same manner of previous test. There was at least 3 minutes interval before and after each stimulation.

Supplementary Figures and Legends S1-S8

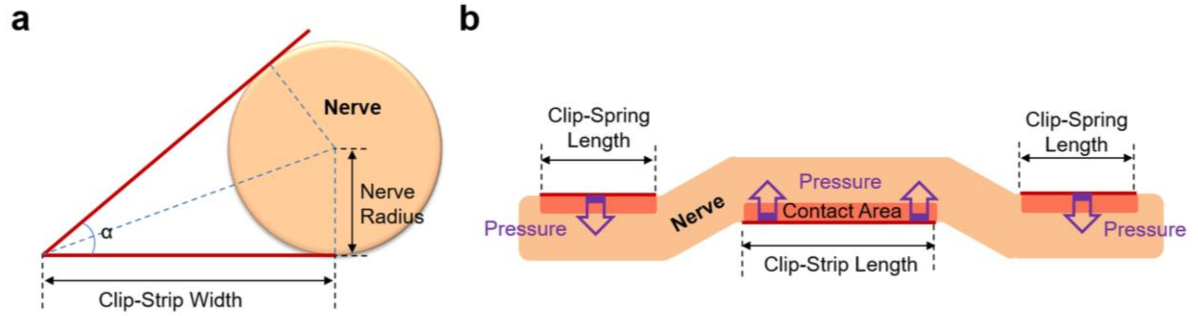

Figure S1. Design of flexible neural clip (FNC) interface. a) Schematic diagram of opening clip head for implantation of a nerve. b) Schematic diagram of cross-section view after the implantation.

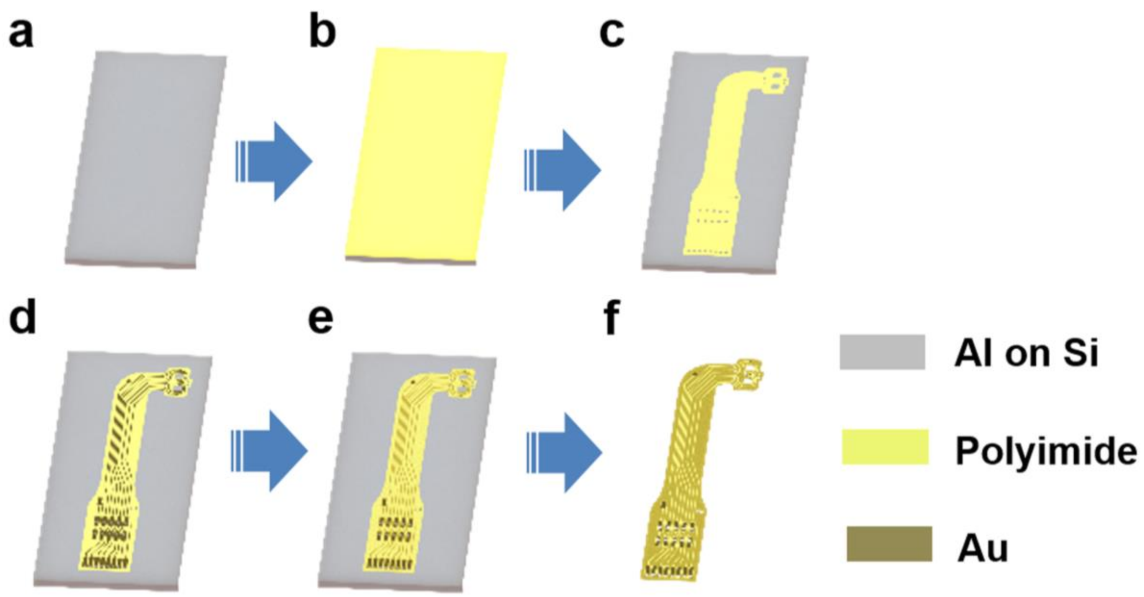

Figure S2. Fabrication process for a flexible neural clip (FNC) interface. a) A 1 μm thick aluminum (Al) layer was evaporated onto the silicon substrate by physical vapor deposition. b) A 8 μm base layer of photosensitive polyimide (Durimide 7505, Fujifilm, Japan) was spun onto the Al coated substrate and c) patterned/cured the bottom layer of the FNC. d) A layer of 20 nm chrome (Cr) and a 250 nm gold layer was deposited and patterned, subsequently by a lift-off process. e) Another 8 μm top layer of polyimide was spun onto the processed metal layer, and patterned to expose the sensing contacts and connection pads. f) Then, released the device by electrochemical method.

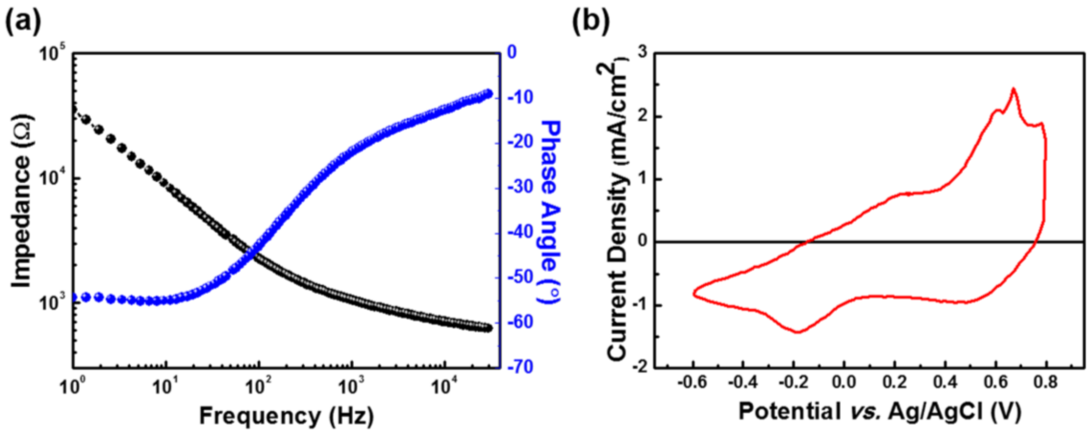

Figure S3. The results of electrochemical characterization of iridium oxide coated electrodes for a) impedance, phase angle, and b) cyclic voltammetry.

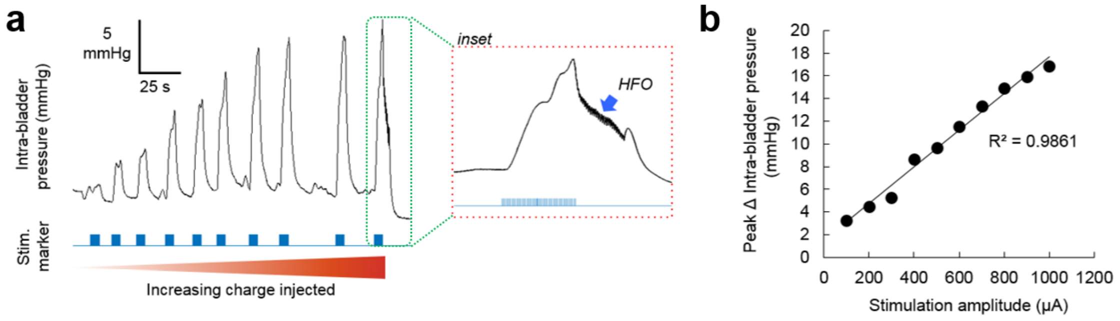

Figure S4. a) Continuous recording of intra-bladder pressure with increasing amount of charge injected via the FNC electrode. Duration of stimulation is indicated by blue stimulation markers below the pressure curve. Inset shows the pressure changes associated with the stimulation trial using the highest amplitude. High frequency oscillations associated (HFO) with voiding response was observed. b) Peak increase in intra-bladder pressure is linearly correlated with amount of stimulation amplitude used.

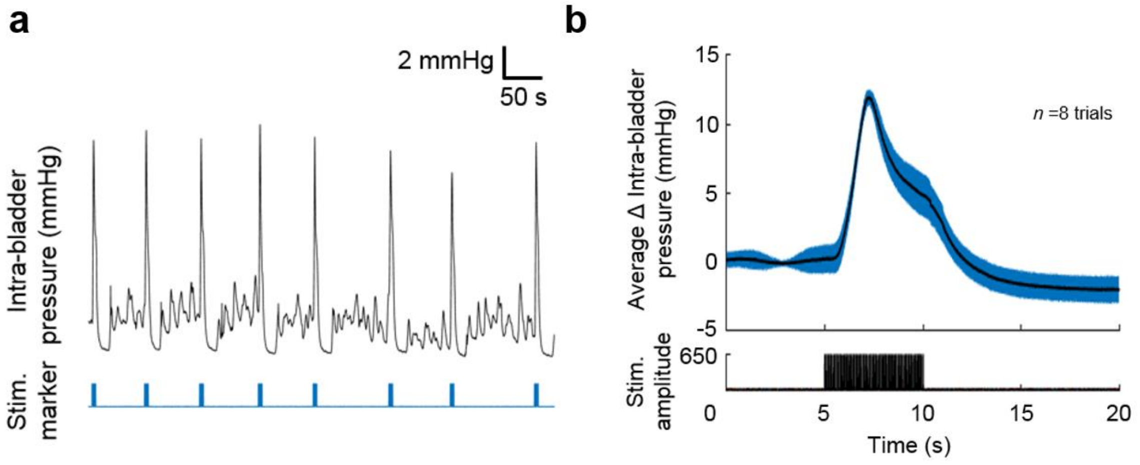

Figure S5. Supra-threshold stimulation of pelvic nerve produced repeatable increases in intra-bladder pressure. a) Continuous recording of intra-bladder pressure (mmHg) during 8 trials of pelvic nerve stimulation at frequency of 10 Hz and amplitude of 650 µA. Voiding responses were observed in all 8 trials. b) Average change in intra-bladder pressure curve from 8 trials of stimulation is indicated by the black curve while the blue shaded region indicates standard deviation.

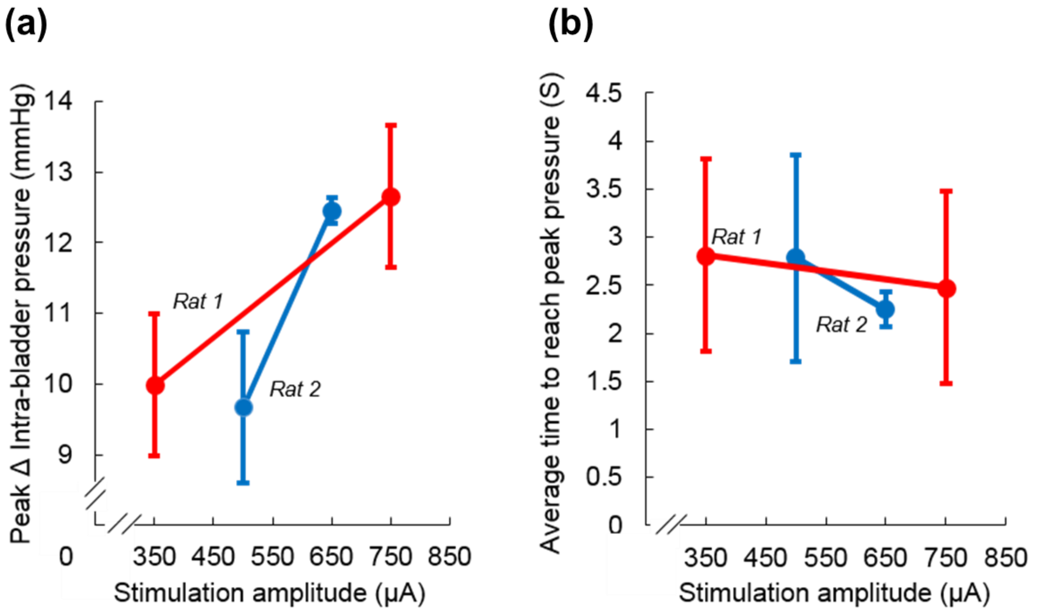

Figure S6. The same clip electrode produced similar responses in different rats for acute pelvic nerve stimulation experiments. Increasing stimulation amplitudes correlated with increasing peak intra-bladder pressure changes a) and faster time to reach peak pressure during stimulation b) in 2 consecutive experiments using the same electrode (N =2 rats).

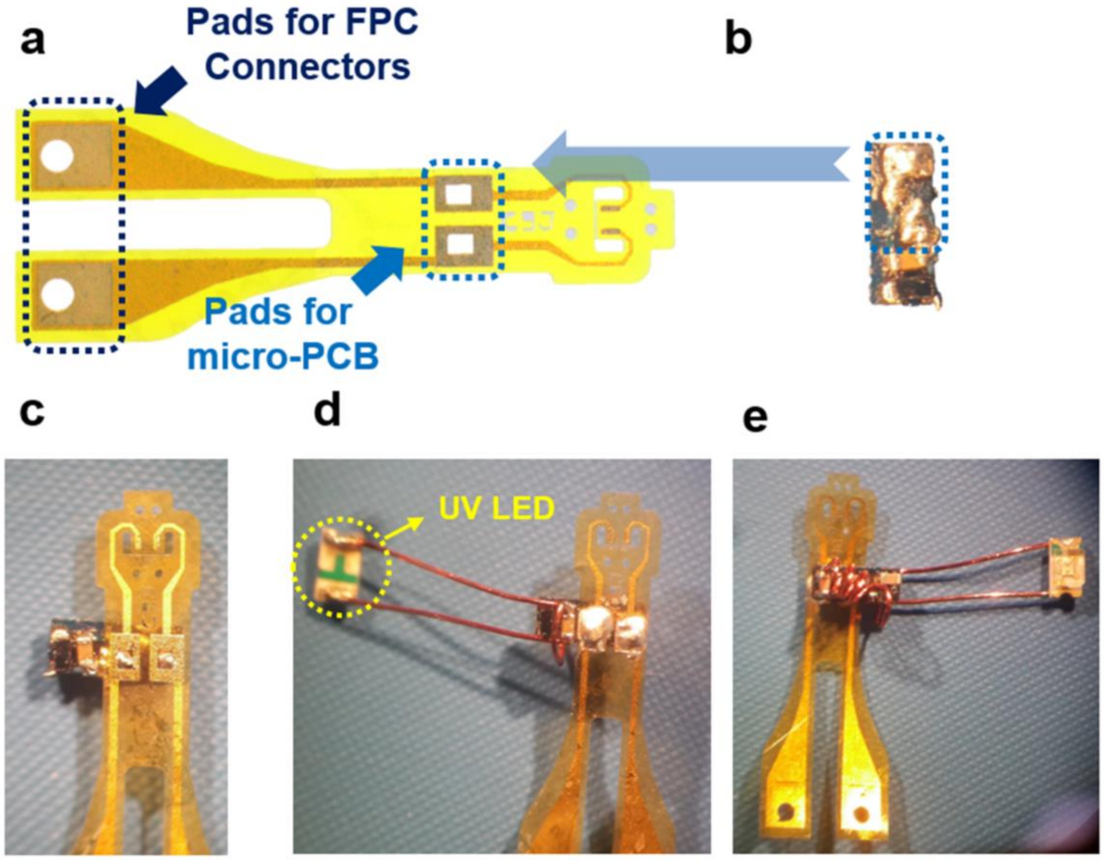

Figure S7. Photomicrograph of a) fabricated active FNC and b) micro-PCB soldered with active components. c) After aligning the FNC on the PCB then, d) conductive epoxy was applied and cured. e) The UV LED and coil were soldered and encapsulated with Kwiksil.

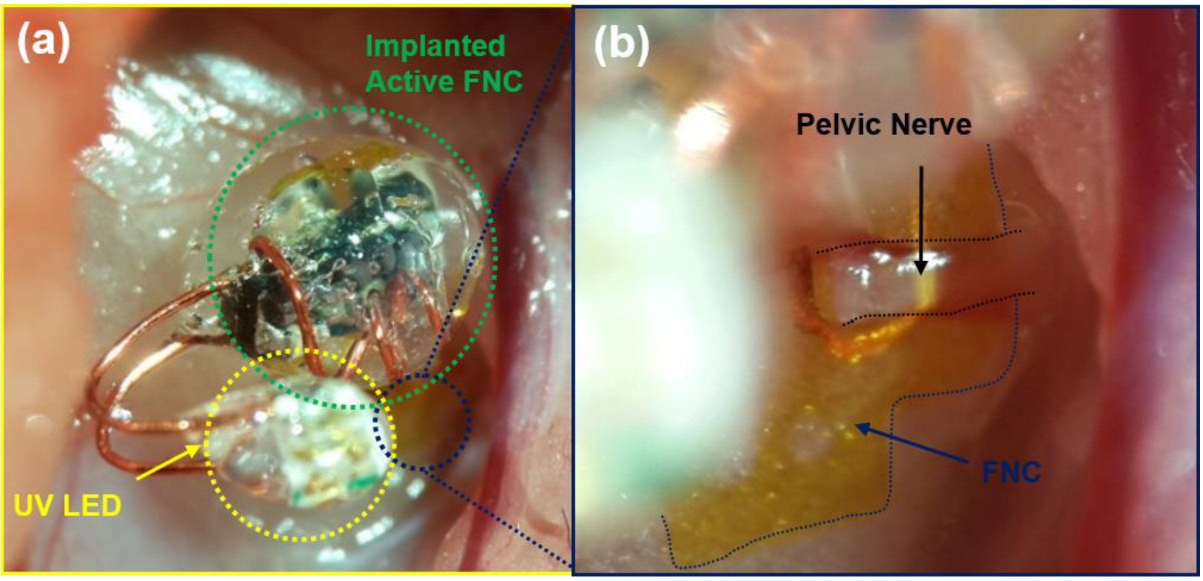

Figure S8. a) A photomicrograph of implanted active flexible neural clip (FNC) interface. b) A magnified photomicrograph of implanted FNC on a pelvic nerve.

References

[1] Z. Xiang, S. Sheshadri, S. H. Lee, J. Wang, N. Xue, N. V. Thakor, S. C. Yen, C. Lee, Advanced Science 2016, 3, 1500386.
